# Supplementary material for: Ferroelectric columnar assemblies from the bowl-to-bowl inversion of aromatic cores
Source: Nat Commun. 2021 Feb 3;12:768. doi: 10.1038/s41467-021-21019-4 (PMC7859410; doi:10.1038/s41467-021-21019-4)
Supplement: Supplementary file 1 — Supplementary Information [file 41467_2021_21019_MOESM1_ESM.pdf]

# Supplementary Information for Ferroelectric Columnar Assemblies Arising from Bowl-to-Bowl Invertible Aromatic Cores

Shunsuke Furukawa,* Jianyun Wu, Masaya Koyama, Keisuke Hayashi, Norihisa Hoshino, Takashi Takeda, Yasutaka Suzuki, Jun Kawamata, Masaichi Saito,* Tomoyuki Akutagawa,*

Table of contents

1. General consideration for the synthesis and characterization ........................ S2
2. Synthetic procedures for all compounds ......................................... S3
3. The optimized structures of triselenasumanene ................................ S6
4. PXRD analysis of **C*n*SS** derivatives............................................ S7
5. POM images of **C*n*SS** derivatives............................................ S9
6. SHG measurements of **C*n*SS** derivatives ...................................... S11
7. Dielectric properties of **C*n*SS** derivatives ...................................... S12
8. Properties of the reference compounds, **C4SeS** and **C6TP** ..................... S15
9. Theoretical calculations of motional freedom ...................................... S17
10. NMR charts ....................................................................... S19

## 1. General consideration for the synthesis and characterization

NMR spectra were recorded on a Bruker AVANCE500 spectrometer ($^1$H NMR, 500 MHz; $^{13}$C NMR, 126 MHz) and a Bruker AVANCE400 spectrometer ($^1$H NMR, 400 MHz; $^{13}$C NMR, 100 MHz). Chemical shifts for $^1$H NMR spectra are reported in parts per million (ppm, δ scale) downfield from tetramethylsilane, and referenced internally to the residual proton in the solvent ($CDCl_3$: δ 7.26). Chemical shifts for $^{13}$C NMR spectra are reported in parts per million (ppm, δ scale) downfield from tetramethylsilane, and are referenced to the $^{13}$C resonance of the NMR solvent ($CDCl_3$: δ 77.00). The data are presented as follows: chemical shift, multiplicity (t = triplet, q = quartet, quint = quintet, m = multiplet and/or multiple resonances), coupling constant in hertz (Hz), and integration. Mass spectra were obtained with a Bruker AutoflexIII (MALDI-TOF) mass spectrometer. Melting points were measured by Yanaco MP-S3 instrument. Column chromatography was performed using Kanto silica gel 60N. Thin layer chromatography was performed using Merck Silica Gel 60 F254, TLC Plates.

## 2. Synthetic procedures for all compounds

### Synthesis of C2SS

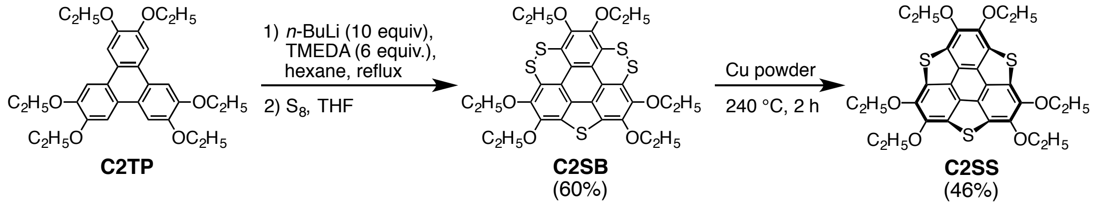


*N*,*N*,*N*′,*N*′-Tetramethylethylenediamine (14.5 mL, 97.3 mmol) was added to a suspension of 2,3,6,7,10,11-hexaethoxytriphenylene (**C2TP**) (8.02 g, 16.3 mmol) in hexane (380 mL). *n*-BuLi in hexane (2.66 M, 62 mL, 165 mmol) was added dropwise to the suspension for 5 min at room temperature, then the mixture was refluxed for 3.5 hours. After cooling to room temperature, volatiles were removed under reduced pressure. THF (300 mL) was added to the resulting solid and then cooled below −60 °C. To the mixture, sulfur powder (8.36 g, 261 mmol) was added in one portion and the mixture was stirred for 30 min at the same temperature. Then the mixture was allowed to warm to room temperature and stirred for 7 hours. After stirring, aqueous $NH_4Cl$ was added to the mixture, and the reaction mixture was extracted with dichloromethane. The extract was dried over anhydrous magnesium sulfate and concentrated under reduced pressure to give a crude product. The products were purified by silica gel column chromatography (hexane/chloroform) to give thienotriphenylenobis(dithiin) **C2SB** (6.31 g, 9.75 mmol, 60%) as a yellow solid.

Copper powder (11.9 g, 187 mmol) was added to **C2SB** (4.04 g, 6.24 mmol), then the mixture was stirred at 240 °C for 2 hours. After cooling to room temperature, dichloromethane was added to the reaction mixture. Insoluble solids were removed by filtration, then volatiles were removed under reduced pressure to obtain crude products. The products were purified by silica gel column chromatography (hexane/dichloromethane) to give **C2SS** (1.69 g, 2.90 mmol, 46%) as a pale yellow solid. Unreacted starting material **2** (1.57 g, 2.43 mmol, 39%) was also recovered.

### Compounds data

**C2SB**: yellow solid, mp. 148–150 °C; $^1$H NMR (400 MHz, $CDCl_3$) δ 1.48–1.53 (m, 18 H), 4.32 (q, *J* = 6.8 Hz, 8H), 4.48 (q, *J* = 6.8 Hz, 4H); $^{13}$C NMR (100 MHz, $CDCl_3$) δ 15.90 ($CH_3$), 15.92 ($CH_3$), 16.10 ($CH_3$), 69.19 ($CH_2$), 70.32 ($CH_2$), 70.62 ($CH_2$), 122.10 (C), 122.48 (C), 124.32 (C), 126.30 (C), 128.49 (C), 129.05 (C), 146.59 (C), 147.07 (C), 148.66 (C); MS (MALDI) *m*/*z* calcd for $C_{30}H_{30}O_6S_5$ ($M^+$) 646.1, found: 646.0; elemental analysis calcd for $C_{30}H_{30}O_6S_5$: C, 55.70; H, 4.67, found: C, 55.79; H, 4.49.

**C2SS**: pale yellow solid, 204-205 °C; $^1$H NMR (400 MHz, $CDCl_3$) δ 1.55 (t, *J* = 6.8 Hz, 18 H), 4.48

(q, $J$ = 6.8 Hz, 12H); $^{13}$C NMR (100 MHz, $CDCl_3$) δ 15.99 ($CH_3$), 69.16 ($CH_2$), 129.97 (C), 130.81 (C), 146.40 (C); MS (MALDI) *m*/*z* calcd for $C_{30}H_{30}O_6S_3$ ($M^+$) 582.1, found: 582.3; elemental analysis calcd for $C_{30}H_{30}O_6S_3$: C, 61.83; H, 5.19, found: C, 61.72; H, 5.03.

**Synthesis of trithiasumanenes bearing long alkyl chains C*n*SS**

**C*n*SS** derivatives ($n$ = 6, 8, 10, and 16) were synthesized by the following general procedure described in the main text (see Methods section).

**Synthesis of C6SS**

For the dealkylation, **C2SS** (150 mg, 0.26 mmol) in dichloromethane (5 mL) was treated with boron tribromide in dichloromethane (1.0 mol $L^{-1}$, 2.6 mL, 2.6 mmol). 1-Bromohexane (0.3 mL, 1.54 mmol), potassium carbonate (305 mg, 2.21 mmol) and DMF (3 mL) were used for the realkylation step. **C6SS** (166 mg, 0.18 mmol, 70%) was obtained as a pale yellow solid. mp. 121 ºC, $^1$H NMR (400 MHz; $CDCl_3$) δ 0.94 (t, $J$ = 7.2 Hz, 18H), 1.41 (br, 24H), 1.58 (quint, $J$ = 7.2 Hz, 12H), 1.90 (quint, $J$ = 6.4 Hz, 12H), 4.40 (t, $J$ = 6.4 Hz, 12H); $^{13}$C NMR (101 MHz; $CDCl_3$) δ 14.21 ($CH_3$), 22.81 ($CH_2$), 25.89 ($CH_2$), 30.38 ($CH_2$), 31.81 ($CH_2$), 73.71 ($CH_2$), 130.26 (C), 130.97 (C), 146.76 (C); MS (MALDI) *m*/*z* calcd for $C_{54}H_{78}O_6S_3$ ($M^+$) 918.5, found: 919.0; elemental analysis calcd for $C_{66}H_{102}O_6S_3$: C, 70.54; H, 8.55, found: C, 70.14; H, 8.55.

**Synthesis of C8SS**

For the dealkylation, **C2SS** (150 mg, 0.26 mmol) in dichloromethane (5 mL) was treated with boron tribromide in dichloromethane (1.0 mol $L^{-1}$, 2.6 mL, 2.6 mmol). 1-Bromooctane (0.35 mL, 2.02 mmol), potassium carbonate (350 mg, 2.54 mmol) and DMF (3 mL) were used for the realkylation step. **C8SS** (117 mg, 0.11 mmol, 42%) was obtained as a pale yellow solid. mp. 92.5 ºC, $^1$H NMR (400 MHz; $CDCl_3$) δ 0.93 (t, 18H), 1.43 (br, 48H), 1.62 (quint, $J$ = 7.2 Hz, 12H), 1.93 (quint, $J$ = 7.2 Hz, 12H), 4.41 (t, $J$ = 6.8 Hz, 12H);$^{13}$C NMR (101 MHz; $CDCl_3$) δ 14.26 ($CH_3$), 22.86 ($CH_2$), 29.27 ($CH_2$), 29.51 ($CH_2$), 29.64 ($CH_2$), 30.47 ($CH_2$), 32.05 ($CH_2$), 73.67 ($CH_2$), 129.96 (C), 130.70 (C), 146.64 (C); MS (MALDI) *m*/*z* calcd for $C_{66}H_{102}O_6S_3$ ($M^+$) 1086.7, found: 1087.2; elemental analysis calcd for $C_{66}H_{102}O_6S_3$: C, 72.88; H, 9.45, found: C, 72.60; H, 9.70.

**Synthesis of C10SS**

For the dealkylation, **C2SS** (200 mg, 0.34 mmol) in dichloromethane (8 mL) was treated with boron tribromide in dichloromethane (1.0 mol $L^{-1}$, 3.5 mL, 3.5 mmol). 1-Bromodecane (0.38 mL, 1.84 mmol), potassium carbonate (260 mg, 1.88 mmol) and DMF (5 mL) were used for the realkylation step. **C10SS** (165 mg, 0.13 mmol, 39%) was obtained as an off-white solid. mp. 83.0 ºC; $^1$H NMR

(500MHz; $CDCl_3$) δ 0.89 (t, $J$ = 7.2 Hz, 18H), 1.29–1.45 (m, 72H), 1.59 (quint, $J$ = 7.2 Hz, 12H), 1.91 (quint, $J$ = 7.2 Hz, 12H), 4.39 (t, $J$ = 6.4 Hz, 12H);$^{13}$C NMR (101 MHz; $CDCl_3$) δ 14.26 ($CH_3$), 22.86 ($CH_2$), 26.28 ($CH_2$), 29.55 ($CH_2$), 29.69 ($CH_2$), 29.82 ($CH_2$), 29.87 ($CH_2$), 30.48 ($CH_2$), 32.11 ($CH_2$), 73.64 ($CH_2$), 129.99 ($CH_3$), 130.72 ($CH_3$), 146.66 ($CH_3$); MS (MALDI) *m/z* calcd for $C_{78}H_{126}O_6S_3$ ($M^+$) 1254.9, found: 1255.5; elemental analysis calcd for $C_{78}H_{126}O_6S_3$: C, 74.59; H, 10.11, found: C, 74.46; H, 10.40.

**Synthesis of C16SS**

For the dealkylation, **C2SS** (194 mg, 0.33 mmol) in dichloromethane (8 mL) was treated with boron tribromide in dichloromethane (1.0 mol $L^{-1}$, 3.5 mL, 3.5 mmol). 1-Bromohexadecane (0.47 mL, 1.54 mmol), potassium carbonate (186 mg, 1.35 mmol) and DMF (5 mL) were used for the realkylation step. **C16SS** (73 mg, 0.04 mmol, 12%) was obtained as a white solid. mp. 70.0 ºC; $^{1}$H NMR (500MHz; $CDCl_3$) δ 0.88 (t, $J$ = 7.1 Hz, 18H), 1.26–1.45 (m, 144 H), 1.57 (quint, $J$ = 7.6 Hz, 12H), 1.90 (quint, $J$ =7.6 Hz, 12H), 4.39 (t, $J$ = 6.4 Hz, 12H); $^{13}$C NMR (101 MHz; $CDCl_3$) δ 14.26 ($CH_3$), 22.68 ($CH_2$), 22.86 ($CH_2$), 23.02 ($CH_2$), 26.27 ($CH_2$), 26.42 ($CH_2$), 29.54 ($CH_2$), 29.68 ($CH_2$), 29.87 ($CH_2$), 29.91 ($CH_2$), 30.46 ($CH_2$), 31.93 ($CH_2$), 32.10 ($CH_2$), 32.27 ($CH_2$), 73.67 ($CH_2$), 130.14 (C), 130.85(C), 146.72 (C); MS (MALDI) *m/z* calcd for $C_{114}H_{198}O_6S_3$ ($M^+$) 1759.4, found: 1760.4; elemental analysis calcd for $C_{114}H_{198}O_6S_3$: C, 77.75; H, 11.33, found: C, 77.24; H, 11.40.

### 3. The optimized structures of triselenasumanene

The geometry optimization of triselenasumanene was performed using Gaussian 09 program[1] at the B3LYP/cc-pVDZ level of theory.

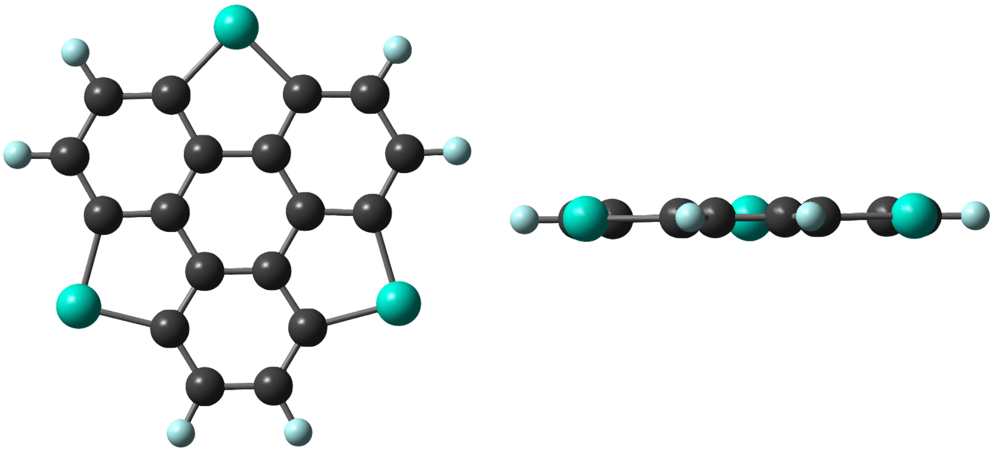

**Supplementary Figure 1.** The optimized structure of triselenasumanene: top view (left) and side view (right).

**Table S1.** Theoretically optimized coordinates of triselenasumanene (Cartesian coordinates in Å)

| atom | *x* | *y* | *z* |
|---|---|---|---|
| C | 0.710683 | 3.612464 | 0 |
| C | -0.710683 | 3.612464 | 0 |
| C | -1.435544 | 2.405058 | 0 |
| C | -0.697602 | 1.221221 | 0 |
| C | 0.697602 | 1.221221 | 0 |
| C | 1.435544 | 2.405058 | 0 |
| Se | 3.329432 | 1.922249 | 0 |
| C | -3.483828 | -1.190763 | 0 |
| C | -2.773144 | -2.421702 | 0 |
| C | -1.365069 | -2.445746 | 0 |
| C | -0.708808 | -1.214751 | 0 |
| C | -1.406409 | -0.00647 | 0 |
| C | -2.800613 | 0.040689 | 0 |
| Se | -3.329432 | 1.922249 | 0 |

| atom | *x* | *y* | *z* |
|---|---|---|---|
| C | 2.773144 | -2.421702 | 0 |
| C | 3.483828 | -1.190763 | 0 |
| C | 2.800613 | 0.040689 | 0 |
| C | 1.406409 | -0.00647 | 0 |
| C | 0.708808 | -1.214751 | 0 |
| C | 1.365069 | -2.445746 | 0 |
| Se | 0 | -3.844497 | 0 |
| H | 3.353812 | -3.34616 | 0 |
| H | 4.574766 | -1.231406 | 0 |
| H | 1.220954 | 4.577566 | 0 |
| H | -1.220954 | 4.577566 | 0 |
| H | -4.574766 | -1.231406 | 0 |
| H | -3.353812 | -3.34616 | 0 |

### 3. PXRD analysis of C*n*SS derivatives

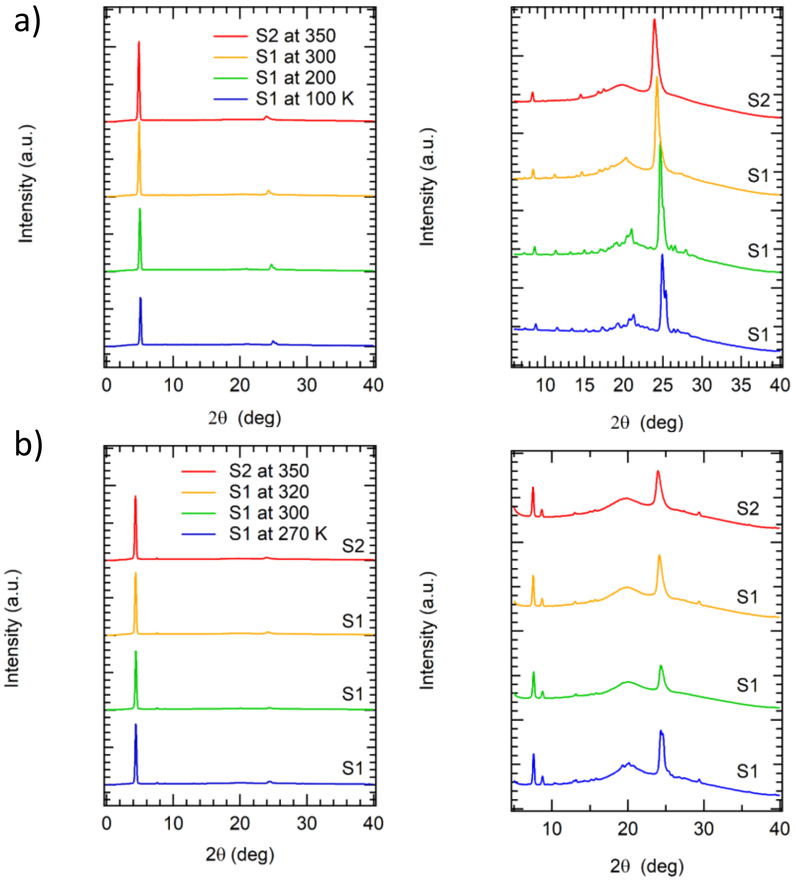


**Supplementary Figure 2.** Temperature-dependent PXRD patterns of a) **C6SS** and b) **C8SS** at S1 and S2 phases with the index assignments of 100, 110, 200, and 001 reflections. Right figure is expanded PXRD pattern at $2\theta$ range from 5 to 40°.

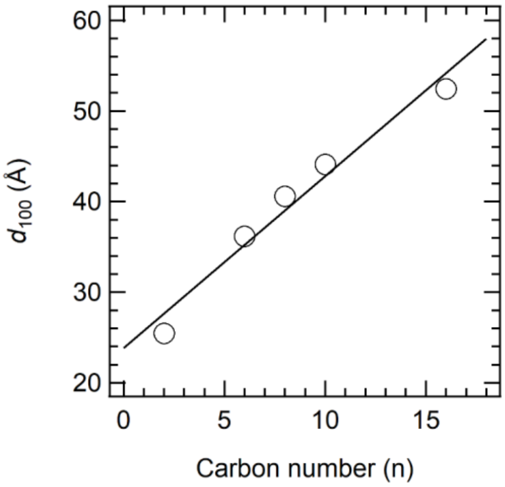

**Supplementary Figure 3.** A linear relationship of and the number of carbon atoms of alkyl chains ($n$ = 2, 6, 8, 10 and 16) and $d_{100}$ spacing.

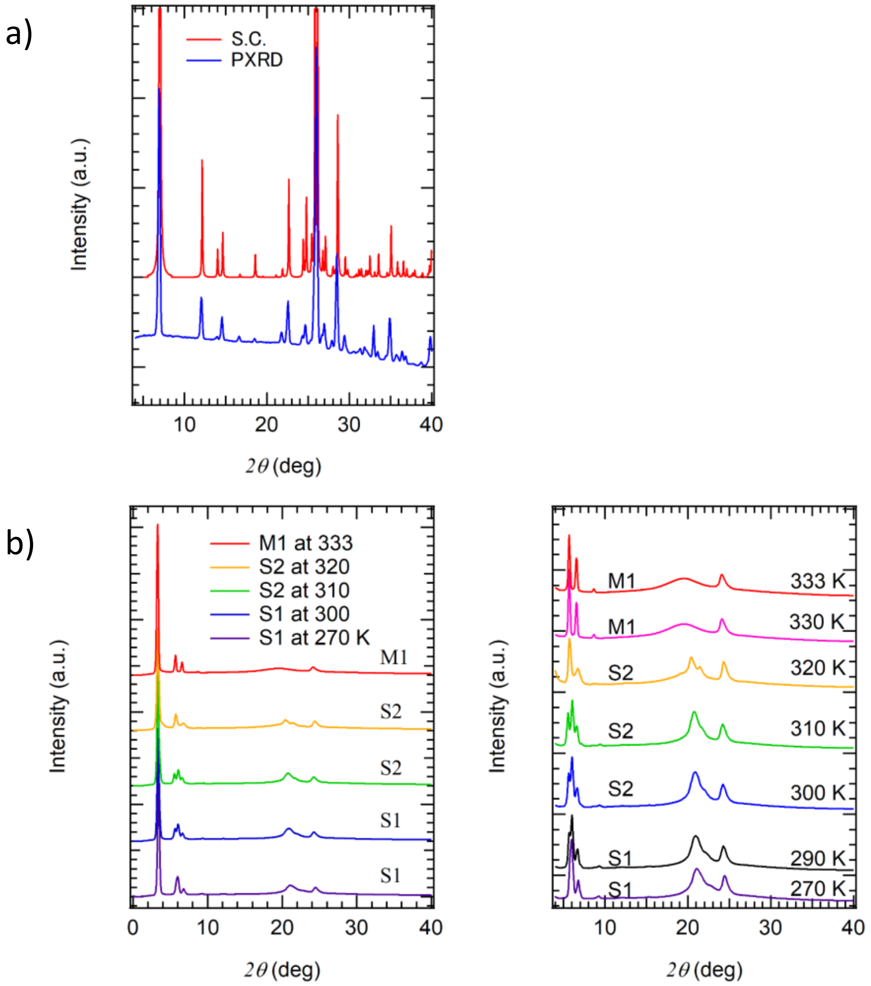


**Supplementary Figure 4.** Crystal structures of **C2SS** and **C16SS**. a) PXRD pattern (blue) and a simulated one (red) based on the single crystal X-ray crystal structural analysis of **C2SS**. b) Temperature dependent changes in the PXRD patterns at S1, S2 and M1 phases. Right figure shows the magnified diffraction patterns in the range of $2\theta$ between 5° and 40°.

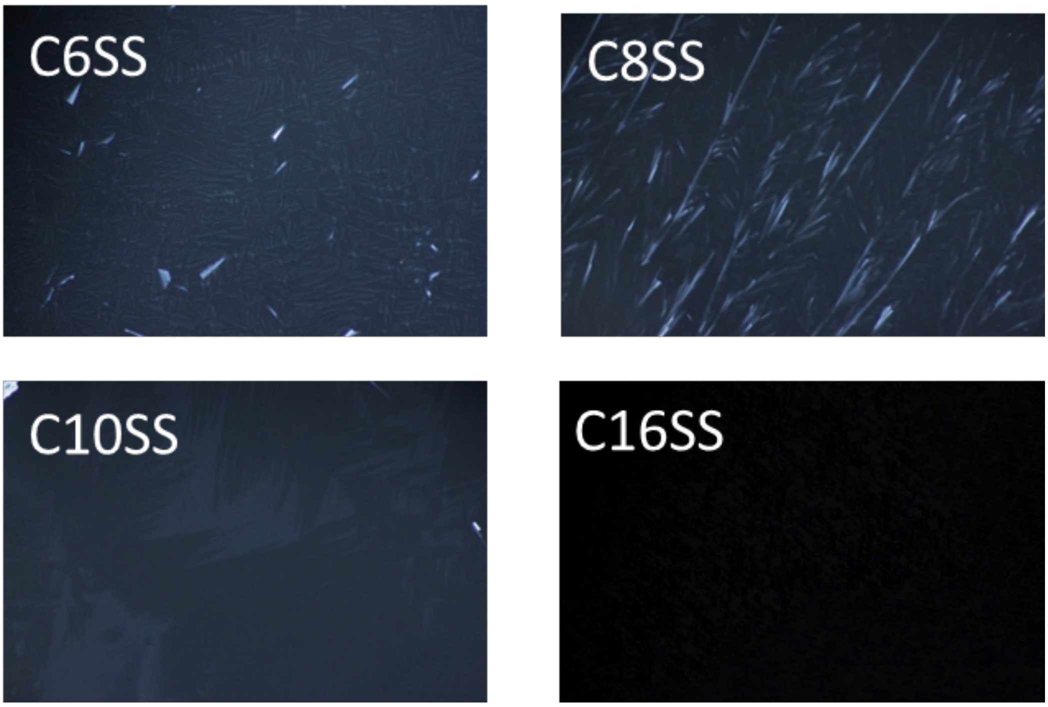

**Supplementary Figure 5.** POM images of S2 phase of **C6SS**, **C8SS**, **C10SS**, and **C16SS**. Dark images under the cross-Nicol optical arrangement were consistent with the homeotropic orientation of each column.

**SHG measurements of C*n*SS.**

The intensity of the SHG activity was measured using the Kurtz powder method. Powder samples were sandwiched between glass plates, with a 58 μm gap, and the temperature-dependent SHG signal was measured five times by comparison with sucrose. Temperature–dependent powder SHG measurement was conducted on glass substrate equipped with temperature controller and the SHG intensity was relative magnitude with sucrose. Although the SHG intensity was not strong due to insufficient polar domain orientation in the absence of the application of electric filed and/or poling procedure, the SHG signal could be observed in the ferroelectric phase without the poling process through the phase transition from non-polar paraelectric to polar ferroelectric phase. A femtosecond pulsed beam (wavelength 1400 nm) from an optical parametric amplifier (Spectra-Physics, OPA-800C) pumped by a beam from a Ti:sapphire regenerative amplifier (Spectra-Physics, Spitfire) was used as the light source. The pulse duration was typically 150–200 fs, and the repetition rate was 1 kHz. The average incident power was 0.2 mW. The incident beam was focused by a plano-convex lens (f = 120 mm). The output SHG signals at wavelength of 700 nm were detected by a photomultiplier tube (Hamamatsu, Model SR250) and processed using a boxcar average (Stanford Research, Model SR250).

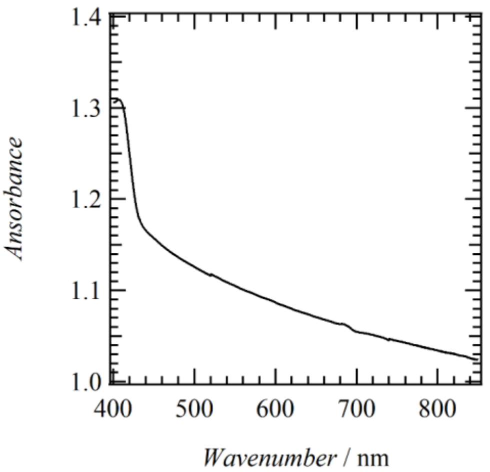


**Supplementary Figure 6.** Solid state diffuse scattering reflection spectra of C10SS at 298 K to examine the resonance energy region for SHG measurement.

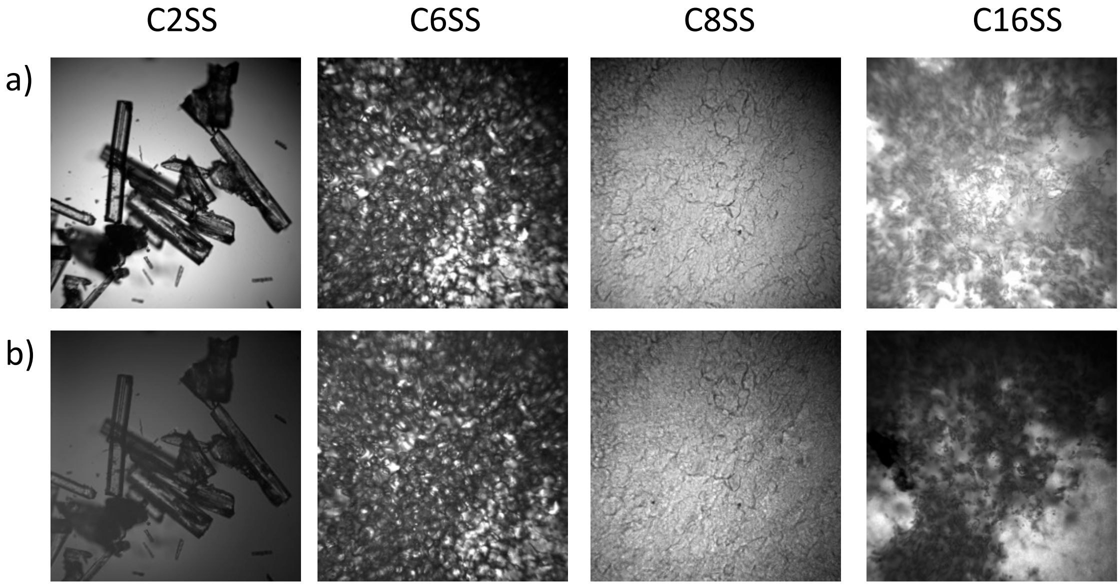


**Supplementary Figure 7.** POM images of **C*n*SS** crystals a) with polarizer and b) without polarizer. The SHG activity was clearly observed at **C2SS** with an intensity of sucrose.

## 4. Dielectric properties of C*n*SS derivatives

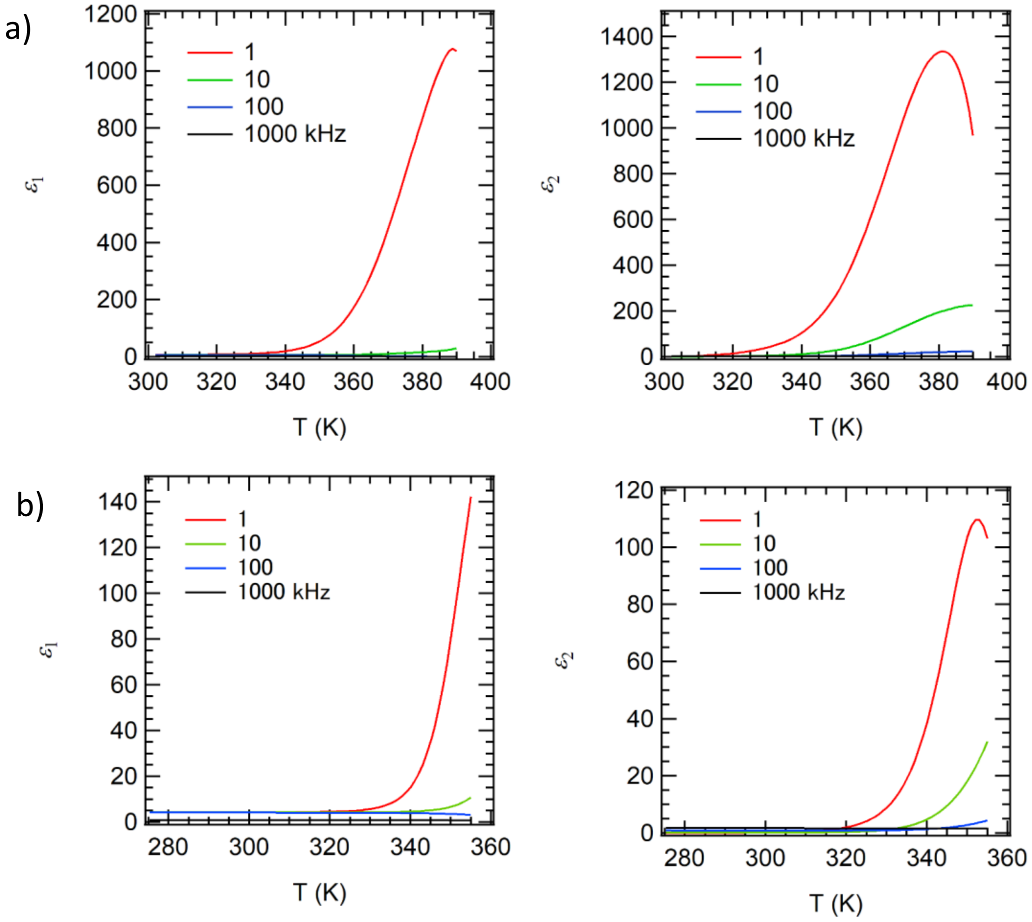


**Supplementary Figure 8.** Dielectric properties of a) **C6SS** and b) **C8SS**. *T*- and *f*-dependent real part $\varepsilon_1$ (left) and imaginary part $\varepsilon_2$ (right) dielectric constants in the heating process.

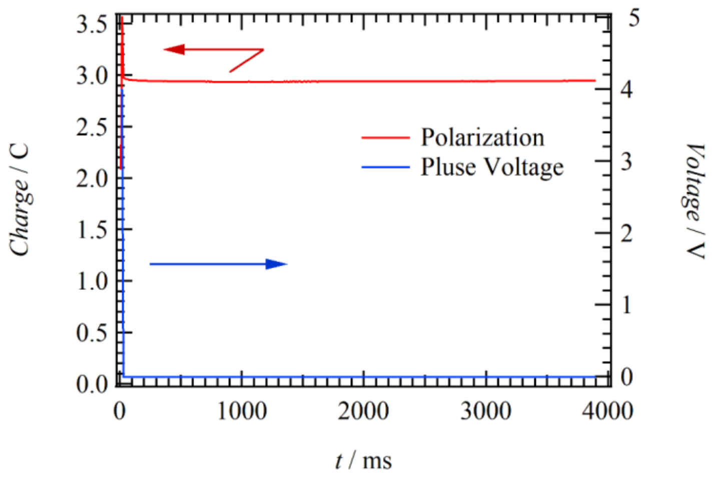


**Supplementary Figure 9.** Time-dependent polarization behavior of **C10SS** at 343K. The pulse voltage was applied at 4 V during 10 msec, then polarization change was monitored.

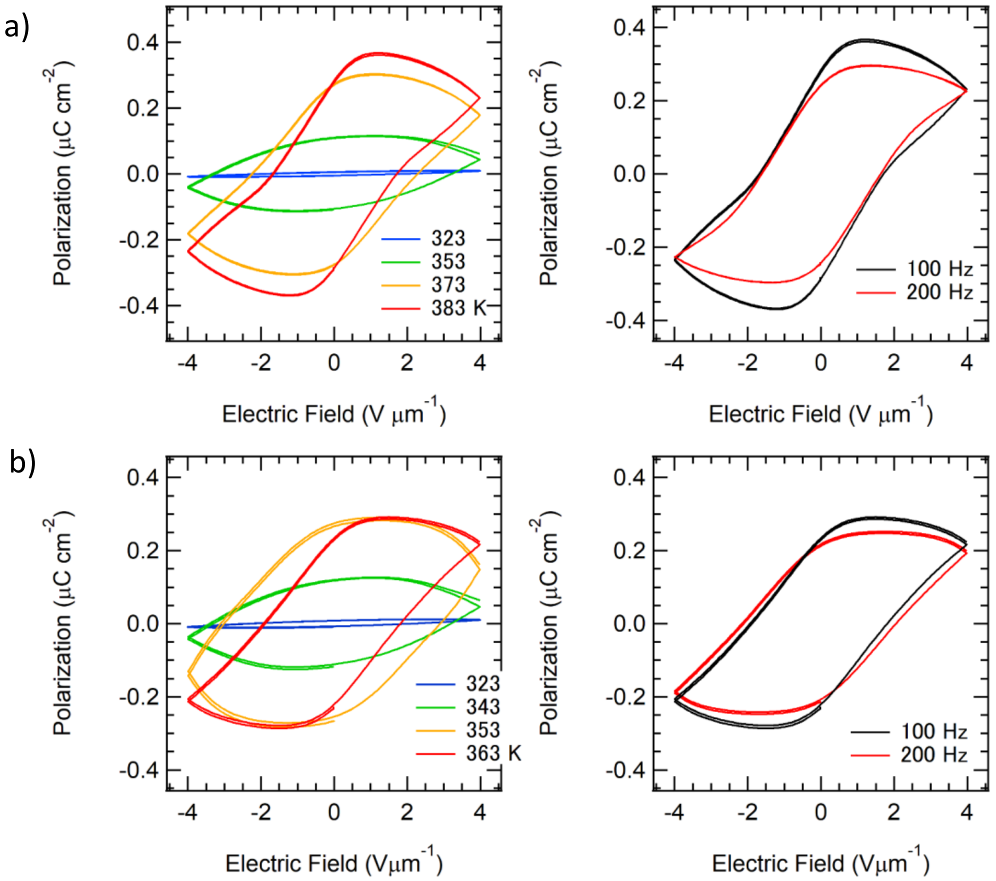


**Supplementary Figure 10.** *T*-dependent (left) and *f*-dependent (right) *P*-*E* hysteresis curves of a) **C6SS** and b) **C8SS**. The *T*-dependent curves were obtained at *f* = 100 Hz. The *f*-dependent curves were obtained at 383 K for **C6SS** and 363 K for **C8SS**.

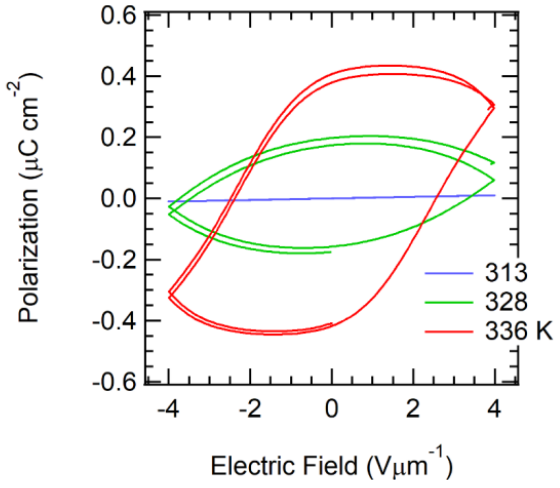


**Supplementary Figure 11.** *T*-dependent *P*-*E* hysteresis curves at *f* = 100 Hz of **C16SS**.

**5. Properties of the reference compounds, C4SeS and C6TP**

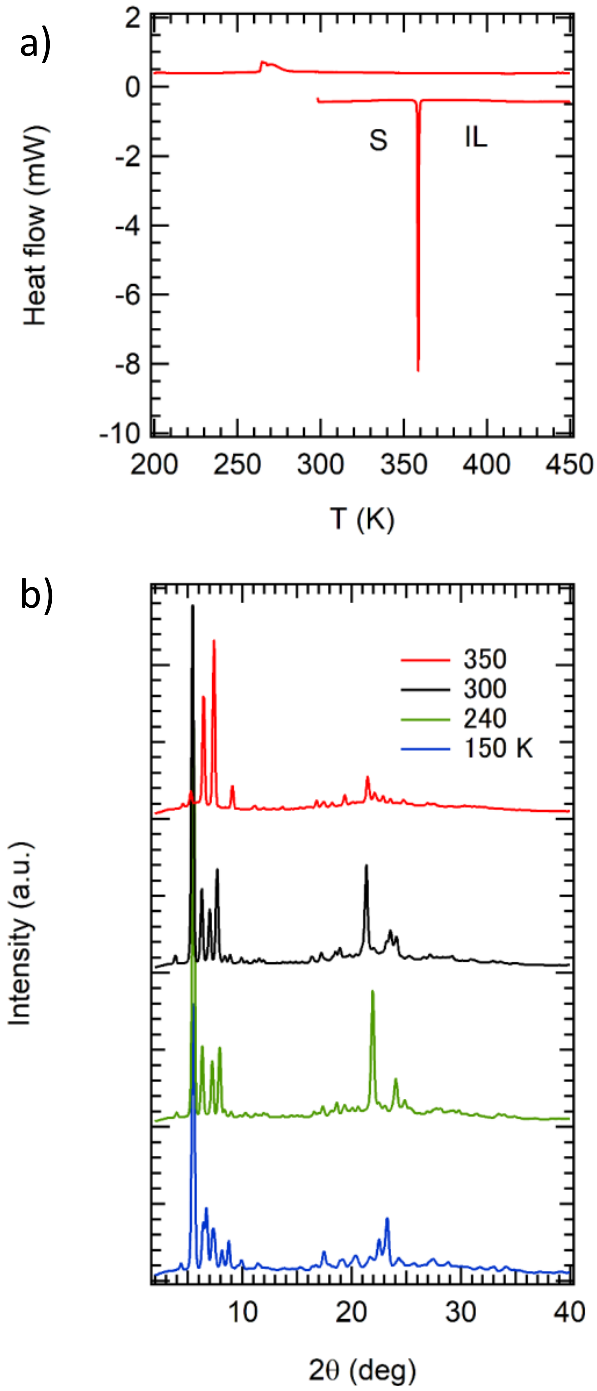


**Supplementary Figure 12.** Phase transition behavior and crystallinity of **C4SeS**. a) DSC chart in the first cycle and b) *T*-dependent PXRD pattern at $T$ = 150, 240, 300, and 350 K.

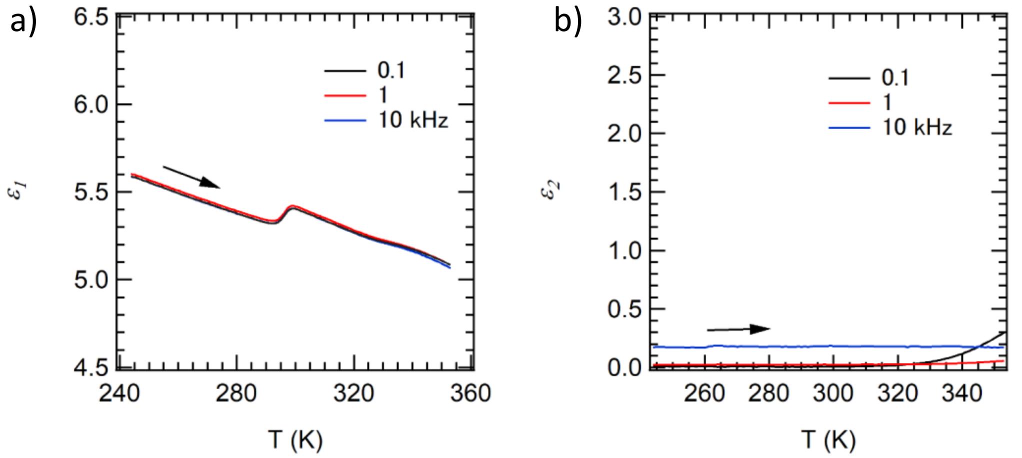


**Supplementary Figure 13.** Temperature and frequency dependent a) real part dielectric constant $\varepsilon_1$ and b) imaginary part dielectric constant $\varepsilon_2$ of **C4SeS** at $f$ = 0.1, 1, and 10 kHz.

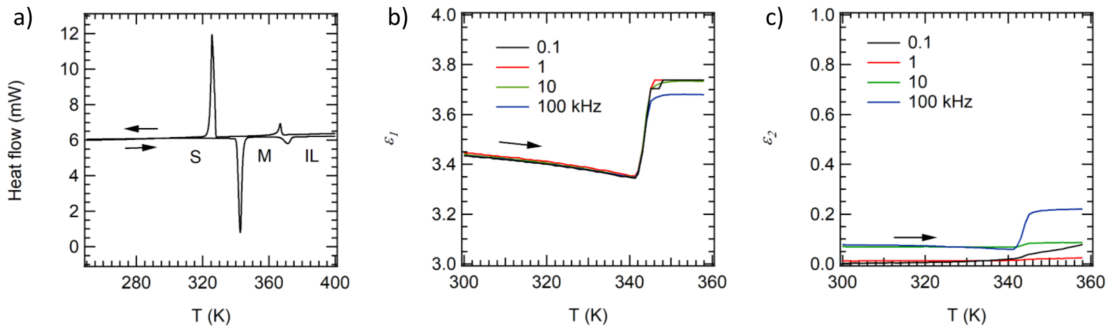


**Supplementary Figure 14.** Phase transition and dielectric behavior of **C6TP**. a) The S-M and M-IL phase transition in DSC chart. $T$- and $f$-dependent b) real part dielectric constant $\varepsilon_1$ and c) imaginary part dielectric constant $\varepsilon_2$ at $f$ = 0.1, 1, 10, and 100 kHz.

**Theoretical calculations of molecular dynamics.**

Theoretical DFT calculation based on a basis function of B3LYP/6-31G(d) was utilized for each **C*n*SS** model calculations. Molecular structures was optimized by fixed angle at C-C-C-S (torsion angle), where the atomic coordinates of central benzene-ring were fixed at the result form the single crystal X-ray structural analysis and the torsion angle of six –$OCH_3$ groups to the benzene-ring was fixed at zero. The skew angel was obtained at **C1SS** dimer model with a fixed inter-dimer distance along the translational operation of the atomic coordinates, where the upper molecule was rotated and obtained the total energy. The energy minimum was defined as a zero for the relative energy scale at each figure.

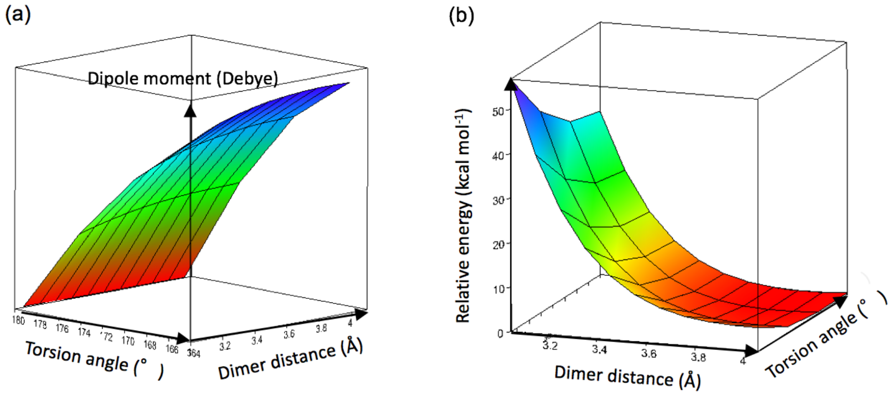


**Supplementary Figure 15.** 3D diagram of stable molecular arrangement. (a) Torsion angle (x) – Dimer distance (y) - dipole moment (z) plot and (b) dimer distance (x) – torsion angle (y) – relative energy (z) plot.

**Supplementary References**


1. Gaussian 09, Revision B.01, M. J. Frisch, G. W. Trucks, H. B. Schlegel, G. E. Scuseria, M. A. Robb, J. R. Cheeseman, G. Scalmani, V. Barone, B. Mennucci, G. A. Petersson, H. Nakatsuji, M. Caricato, X. Li, H. P. Hratchian, A. F. Izmaylov, J. Bloino, G. Zheng, J. L. Sonnenberg, M. Hada, M. Ehara, K. Toyota, R. Fukuda, J. Hasegawa, M. Ishida, T. Nakajima, Y. Honda, O. Kitao, H. Nakai, T. Vreven, J. A. Montgomery, Jr., J. E. Peralta, F. Ogliaro, M. Bearpark, J. J. Heyd, E. Brothers, K. N. Kudin, V. N. Staroverov, T. Keith, R. Kobayashi, J. Normand, K. Raghavachari, A. Rendell, J. C. Burant, S. S. Iyengar, J. Tomasi, M. Cossi, N. Rega, J. M. Millam, M. Klene, J. E. Knox, J. B. Cross, V. Bakken, C. Adamo, J. Jaramillo, R. Gomperts, R. E. Stratmann, O. Yazyev, A. J. Austin, R. Cammi, C. Pomelli, J. W. Ochterski, R. L. Martin, K. Morokuma, V. G.

Zakrzewski, G. A. Voth, P. Salvador, J. J. Dannenberg, S. Dapprich, A. D. Daniels, O. Farkas, J. B. Foresman, J. V. Ortiz, J. Cioslowski, and D. J. Fox, Gaussian, Inc., Wallingford CT, 2010.

## 7. NMR charts

**$^{1}$H NMR spectrum of C2SS in $CDCl_3$**

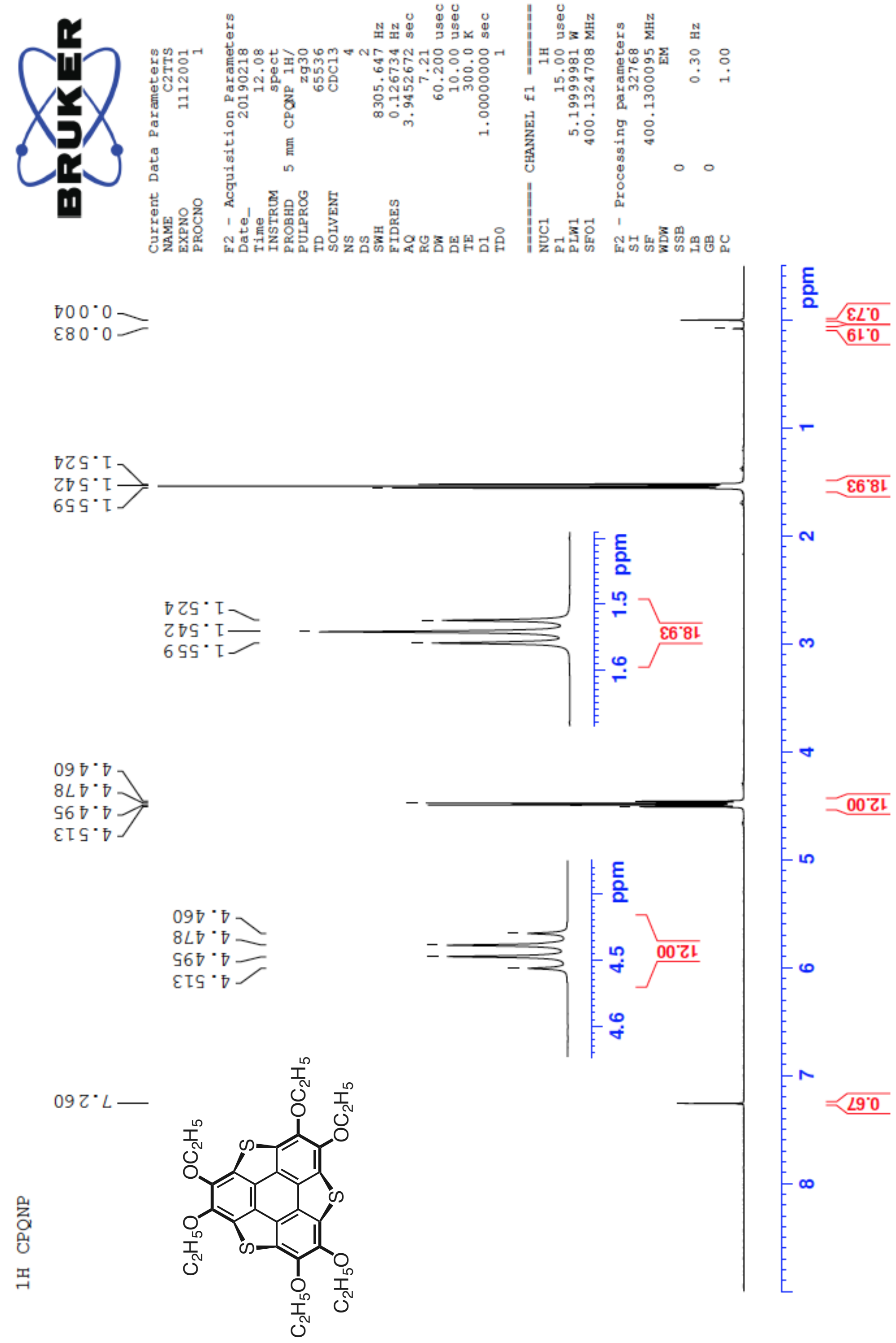

$^{13}$C NMR spectrum of C2SS in $CDCl_3$

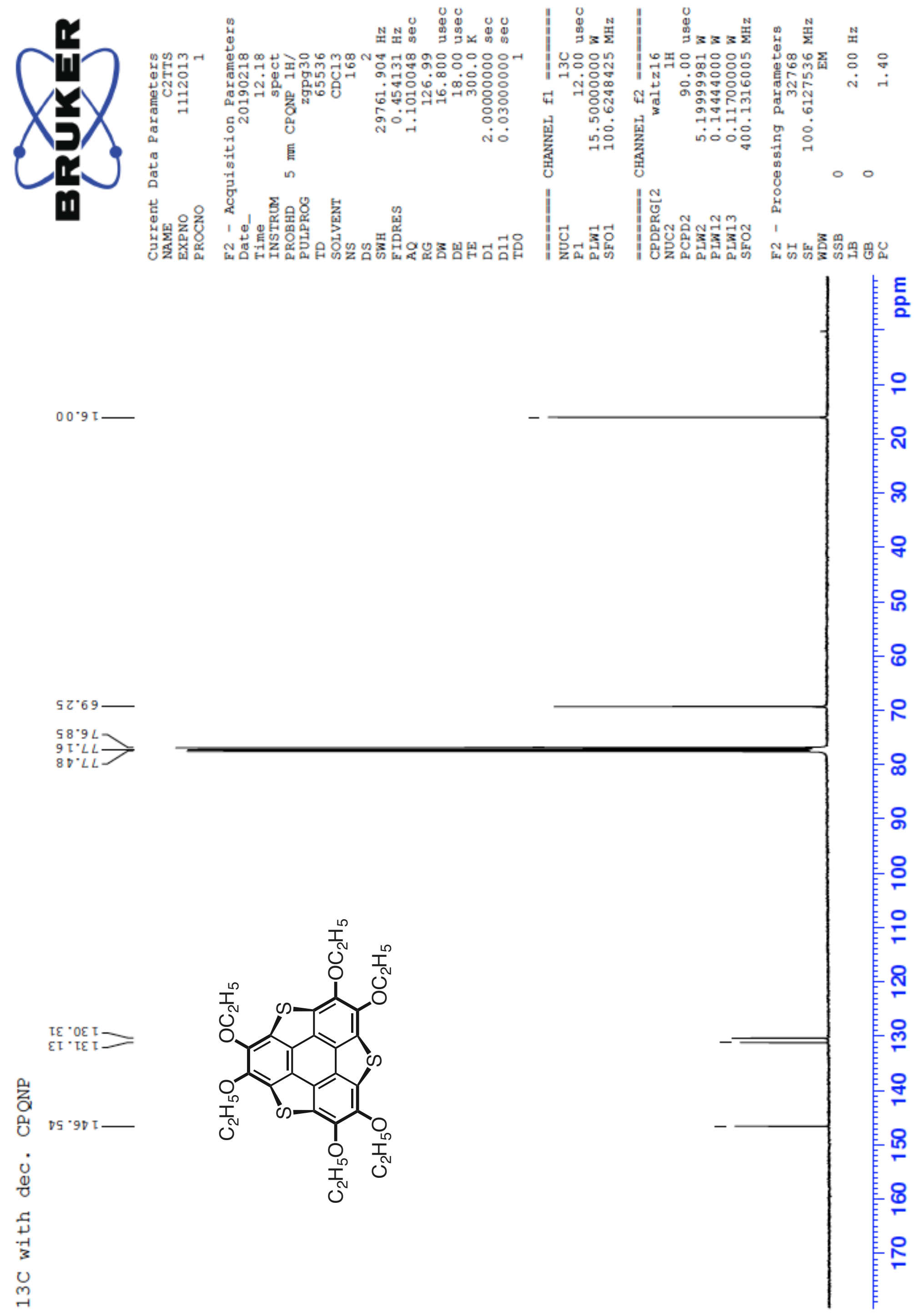

**DEPT-135 of of C2SS in $CDCl_3$**

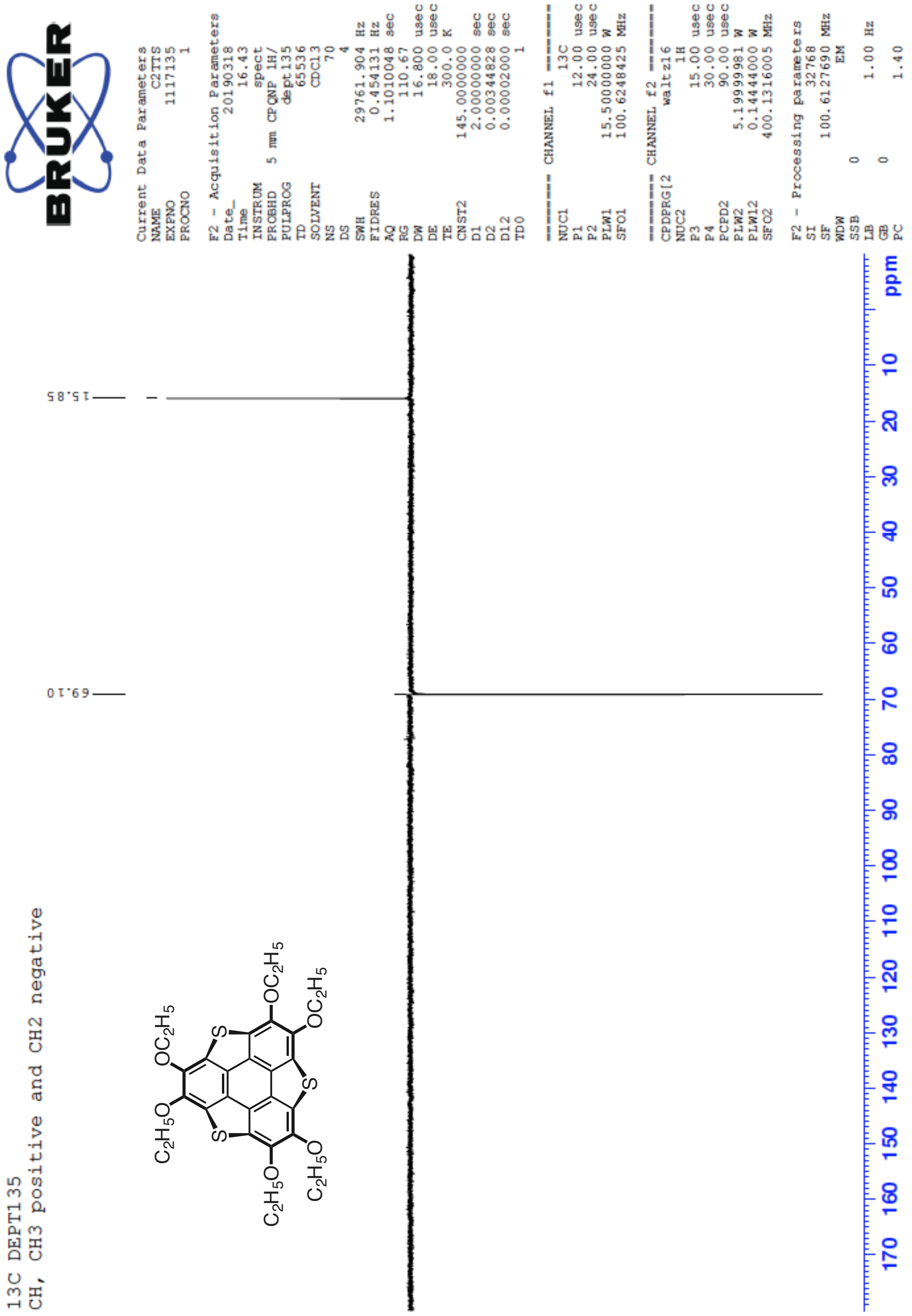

**$^{1}$H NMR spectrum of C6SS in $CDCl_3$**

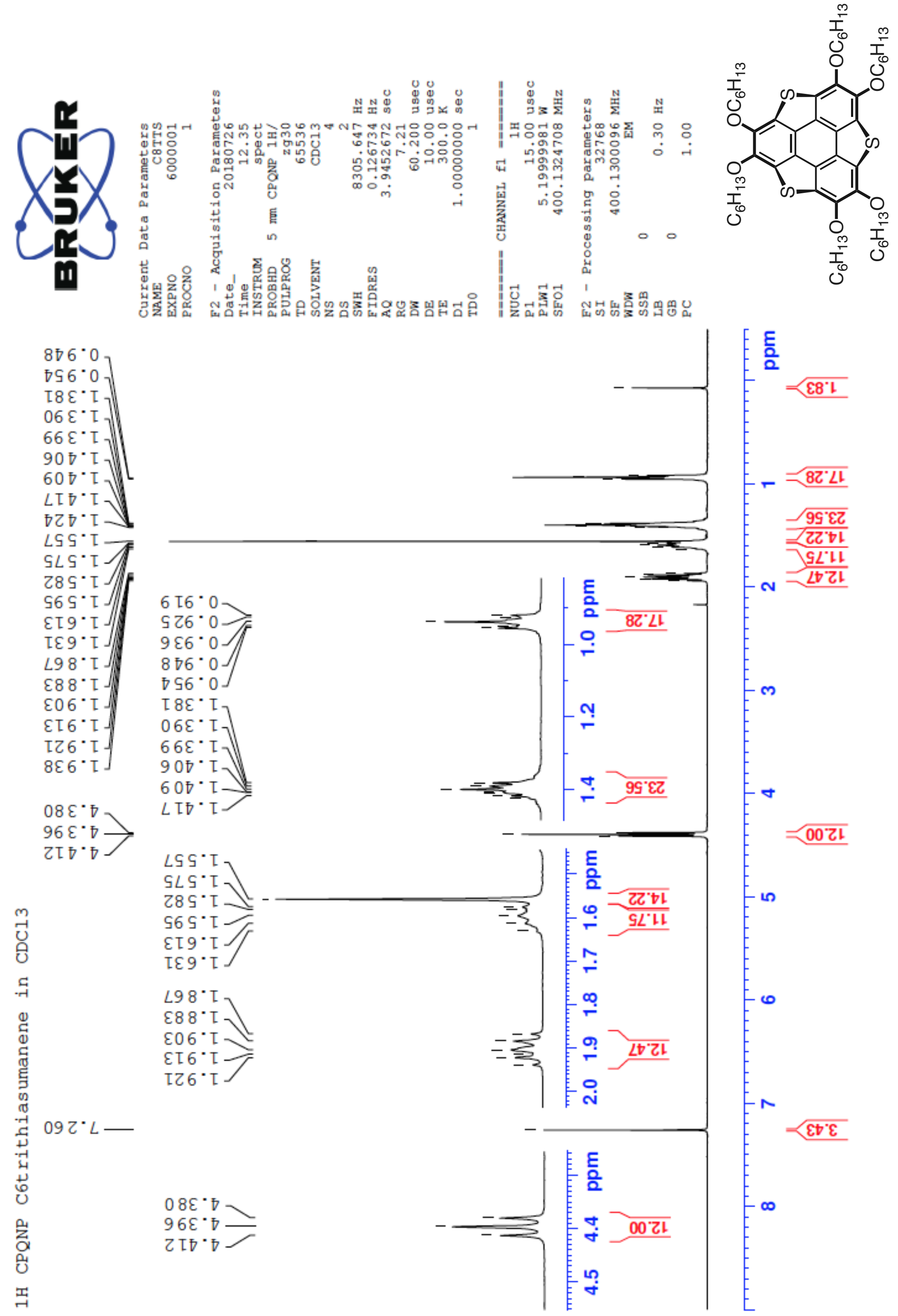

**$^{13}C$ NMR spectrum of C6SS in $CDCl_3$**

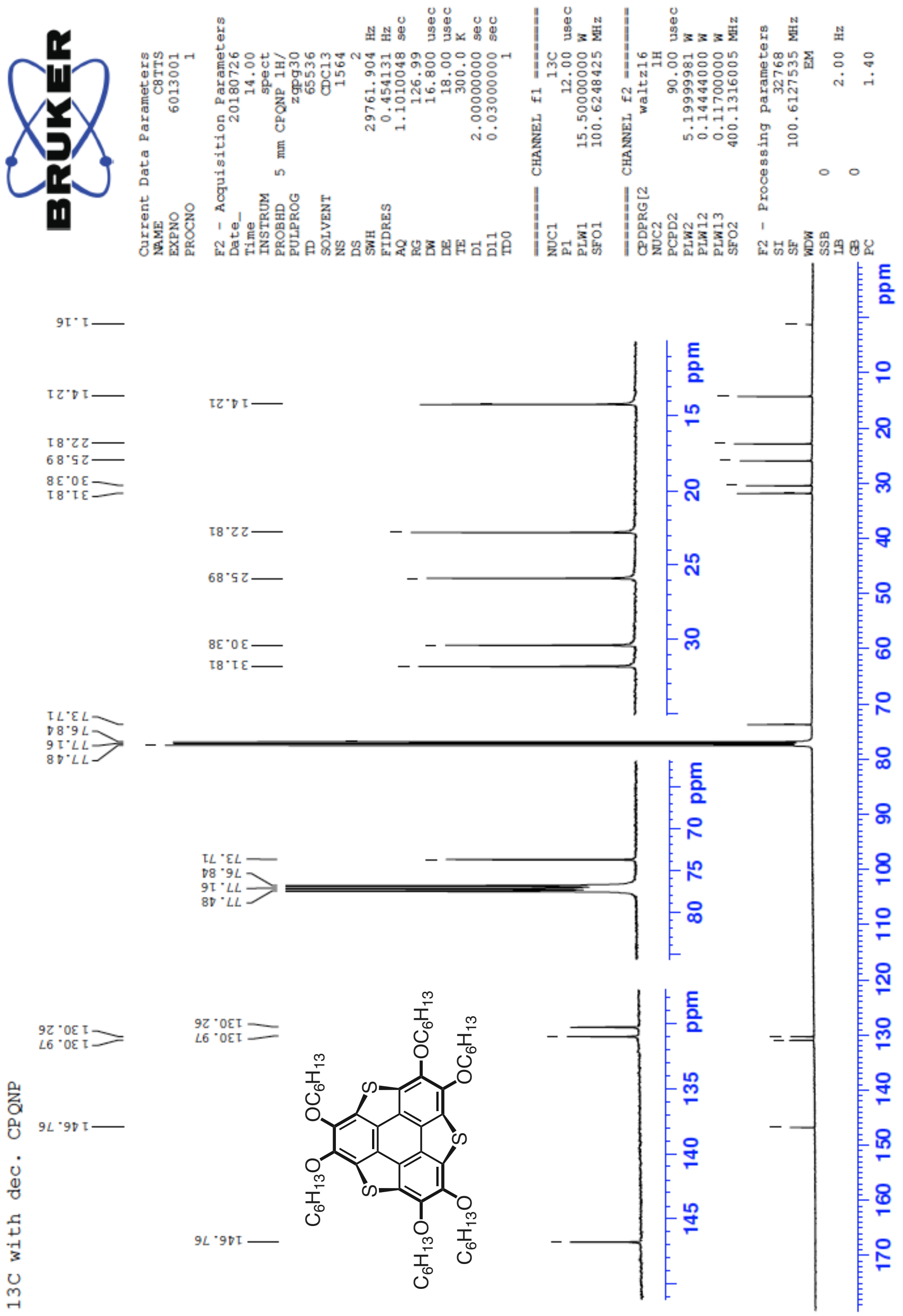

**DEPT-135 of of C6SS in $CDCl_3$**

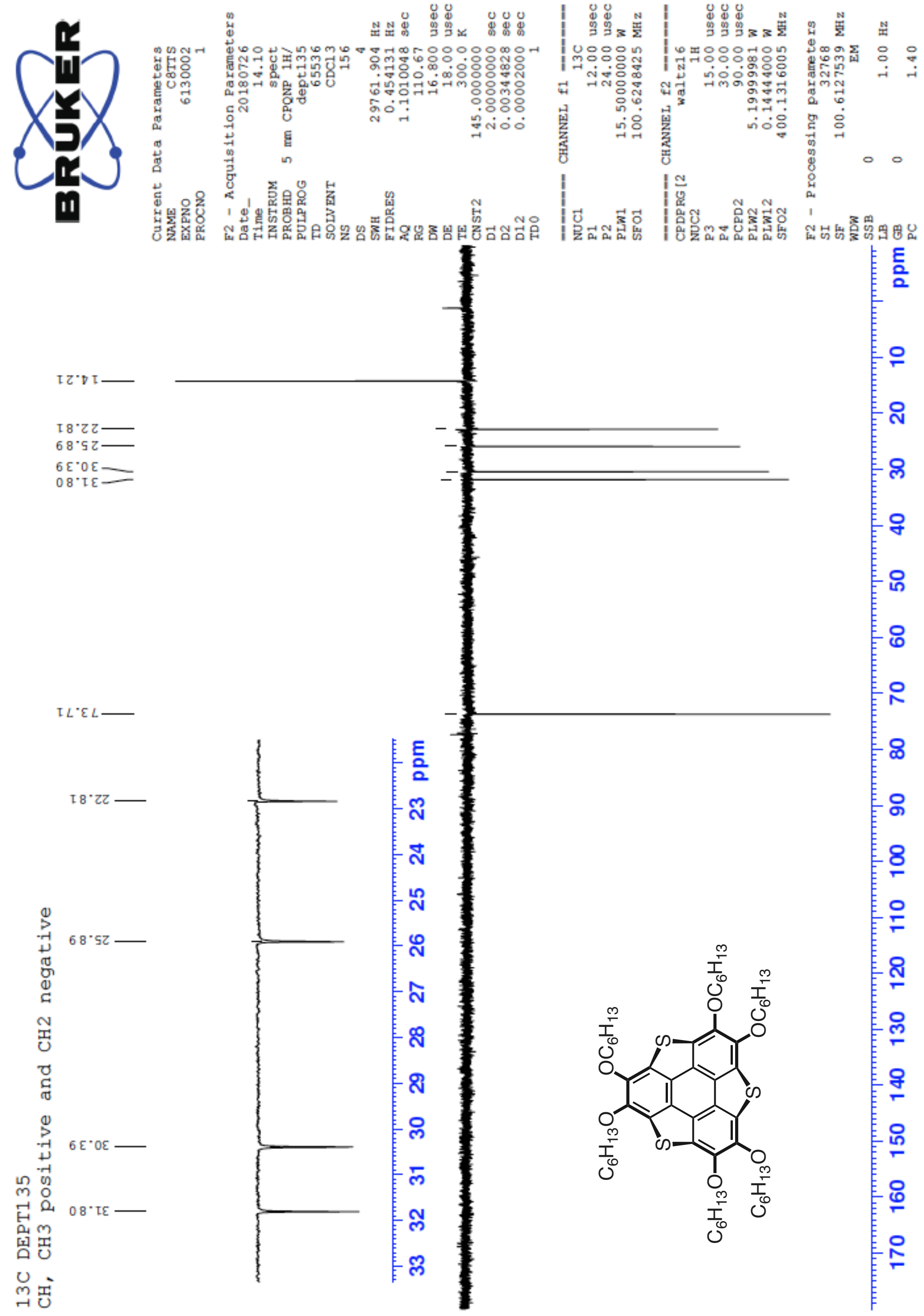

**$^{1}H$ NMR spectrum of C8SS in $CDCl_3$**

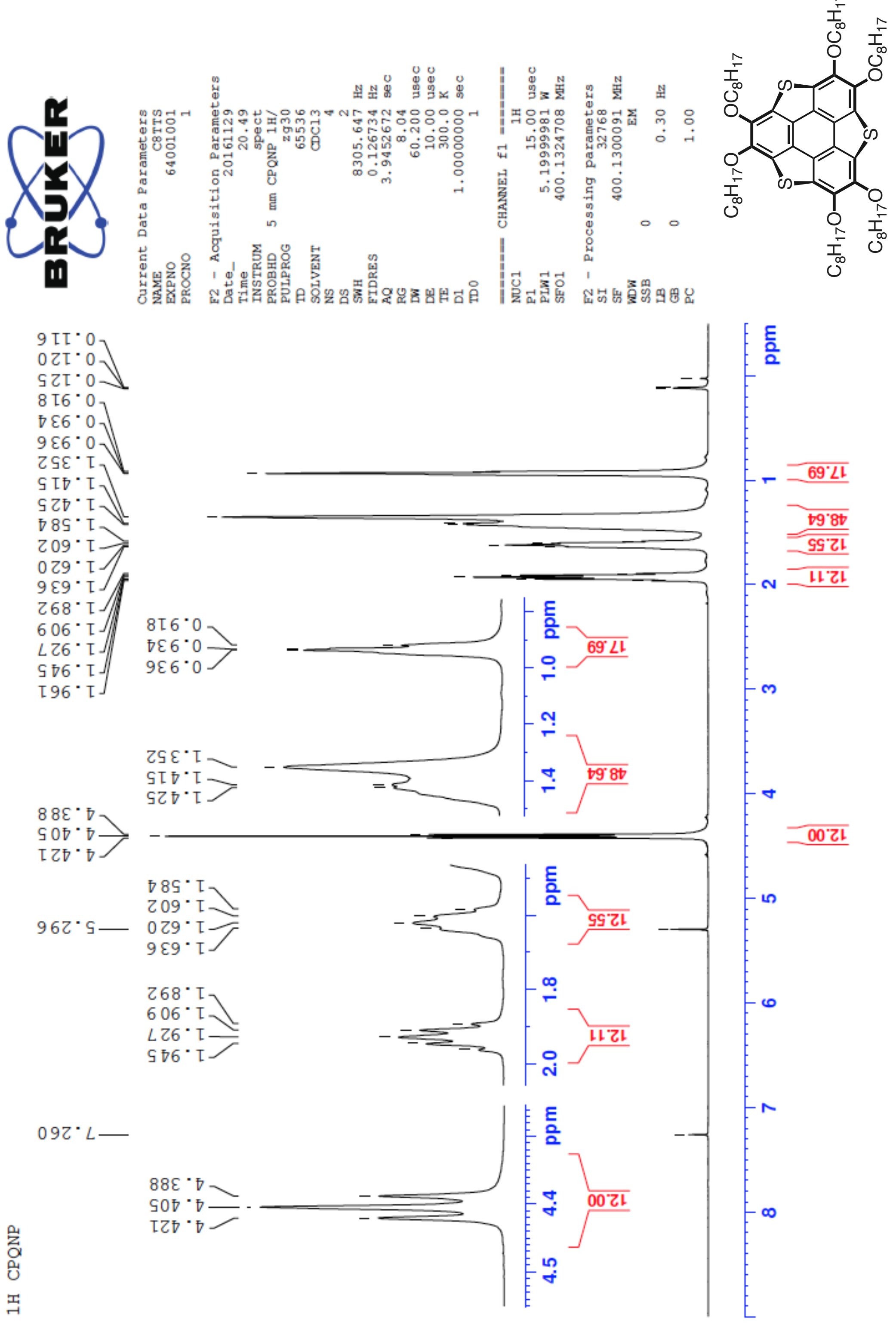

**$^{13}C$ NMR spectrum of C8SS in $CDCl_3$**

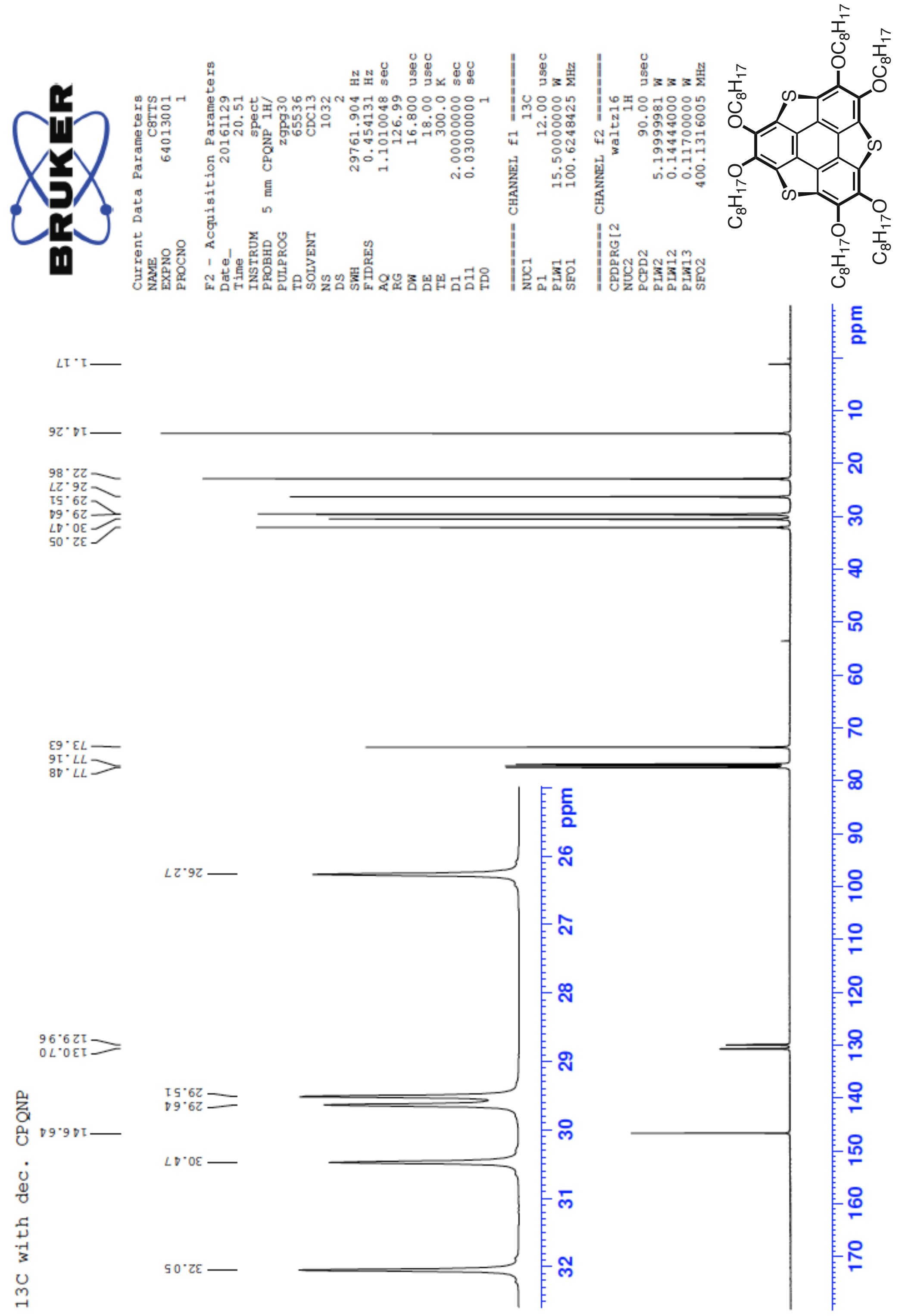

**DEPT-135 of C8SS in $CDCl_3$**

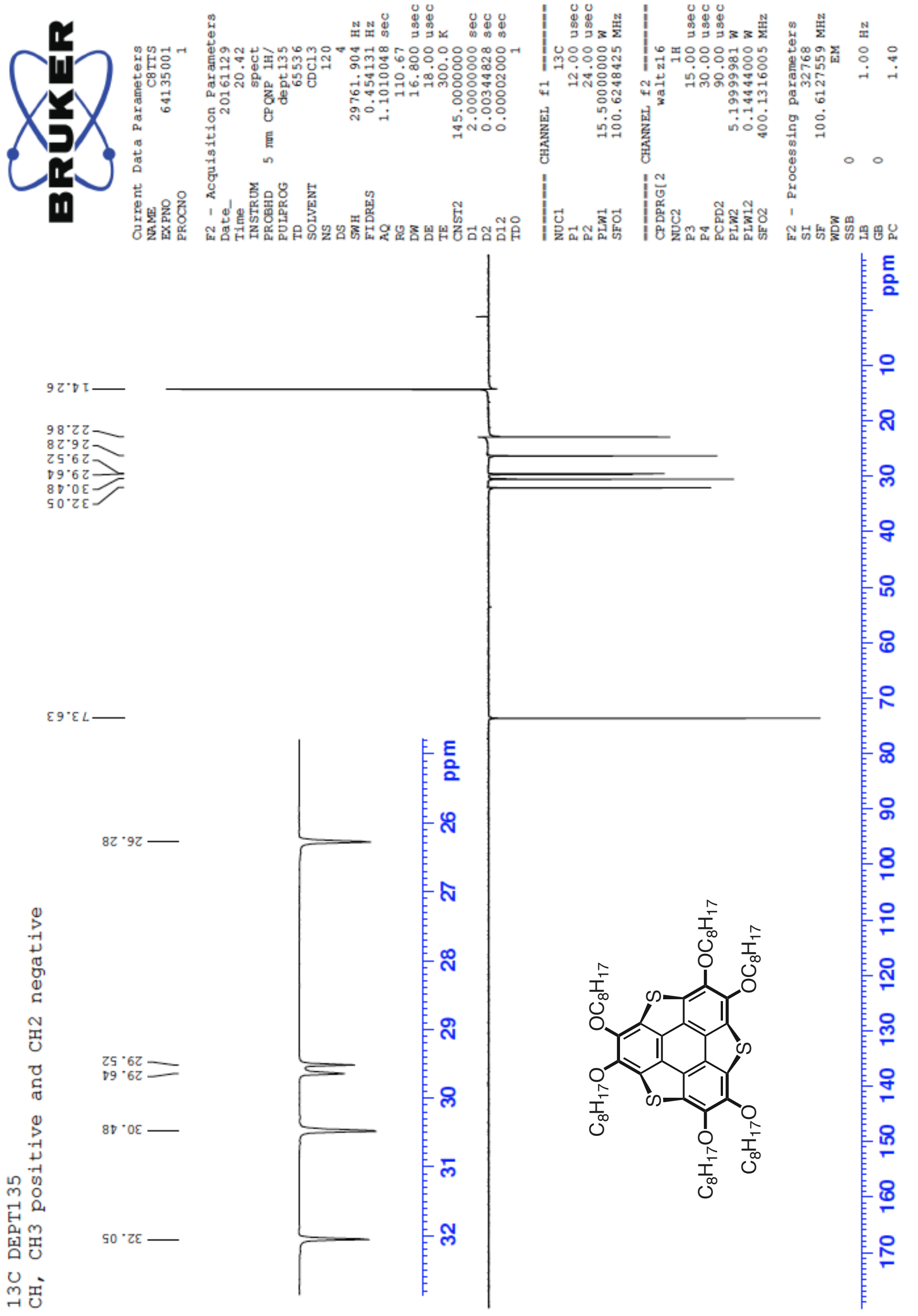

## $^{1}$H NMR spectrum of C10SS in $CDCl_3$

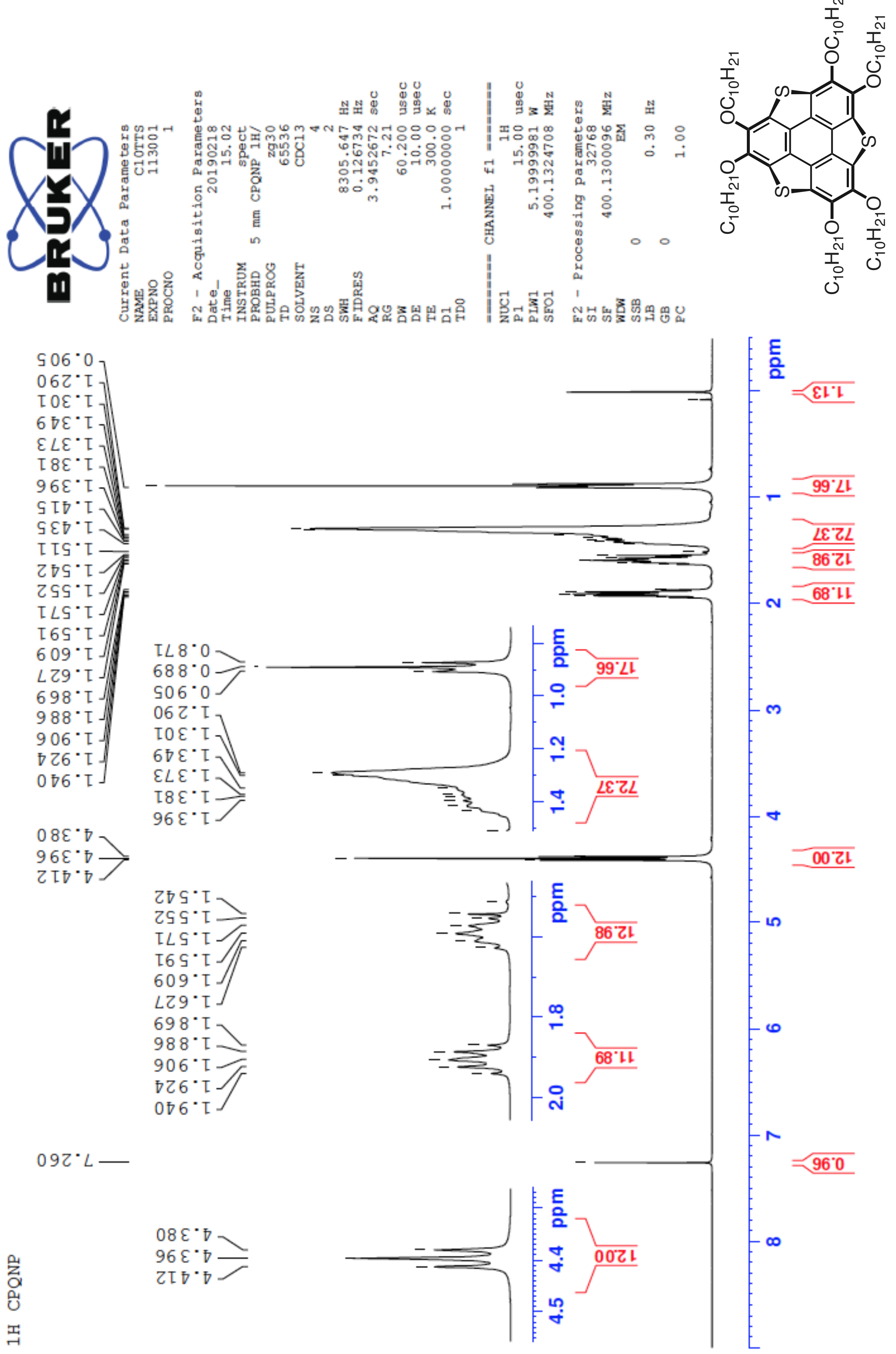


## $^{13}$C NMR spectrum of C10SS in $CDCl_3$

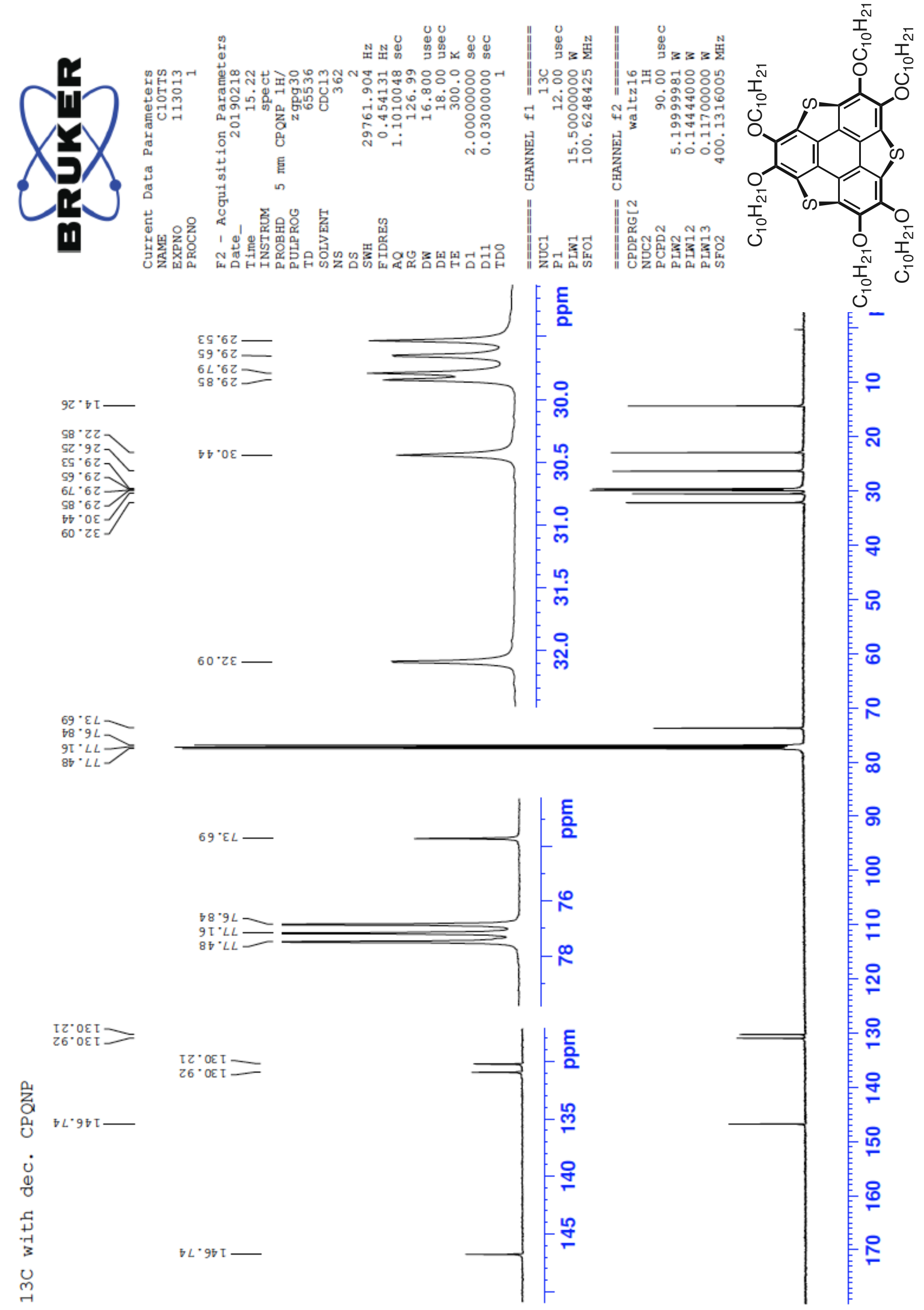

**DEPT-135 of C10SS in $CDCl_3$**

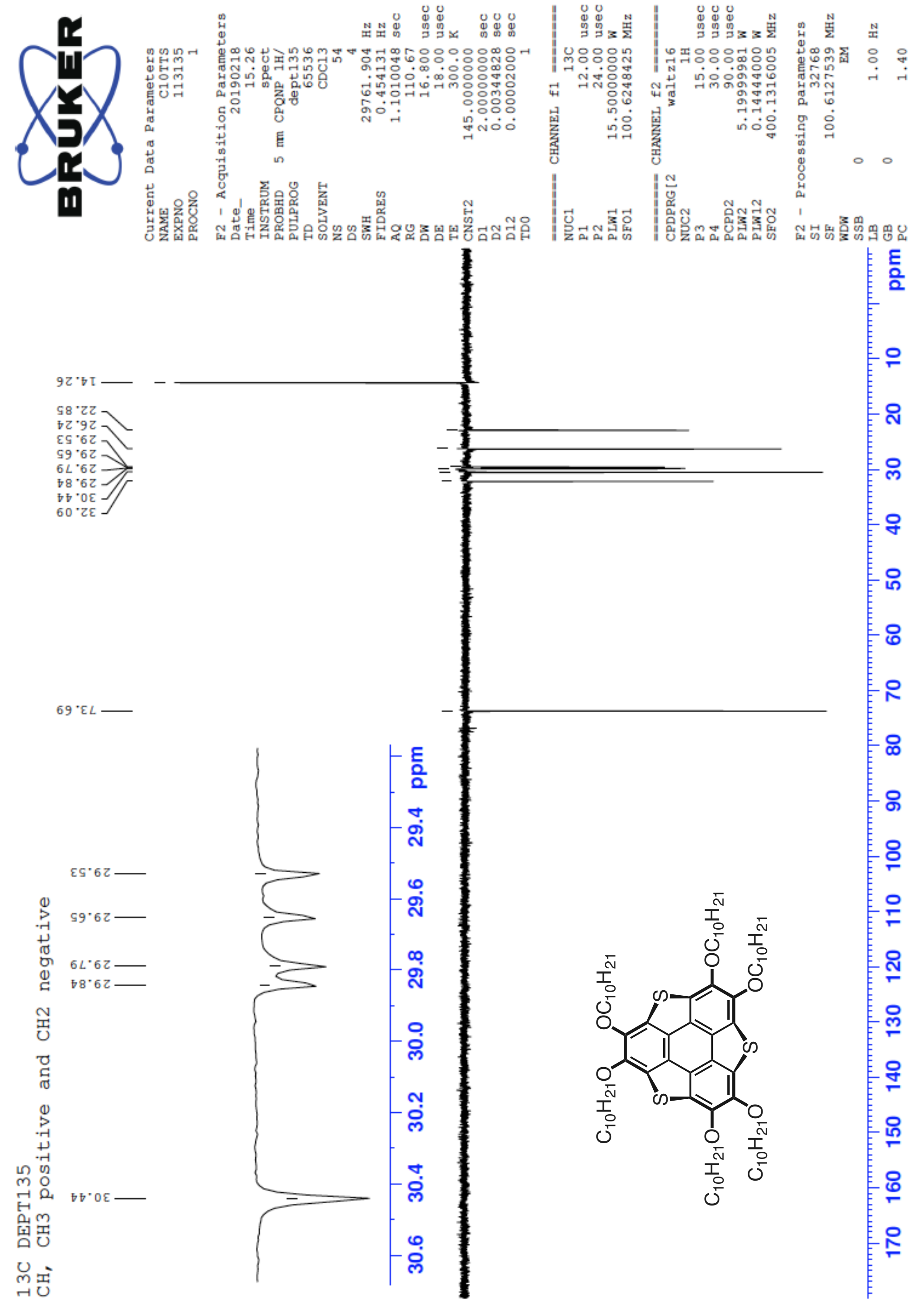


**$^{1}$H NMR spectrum of C16SS in $CDCl_3$**

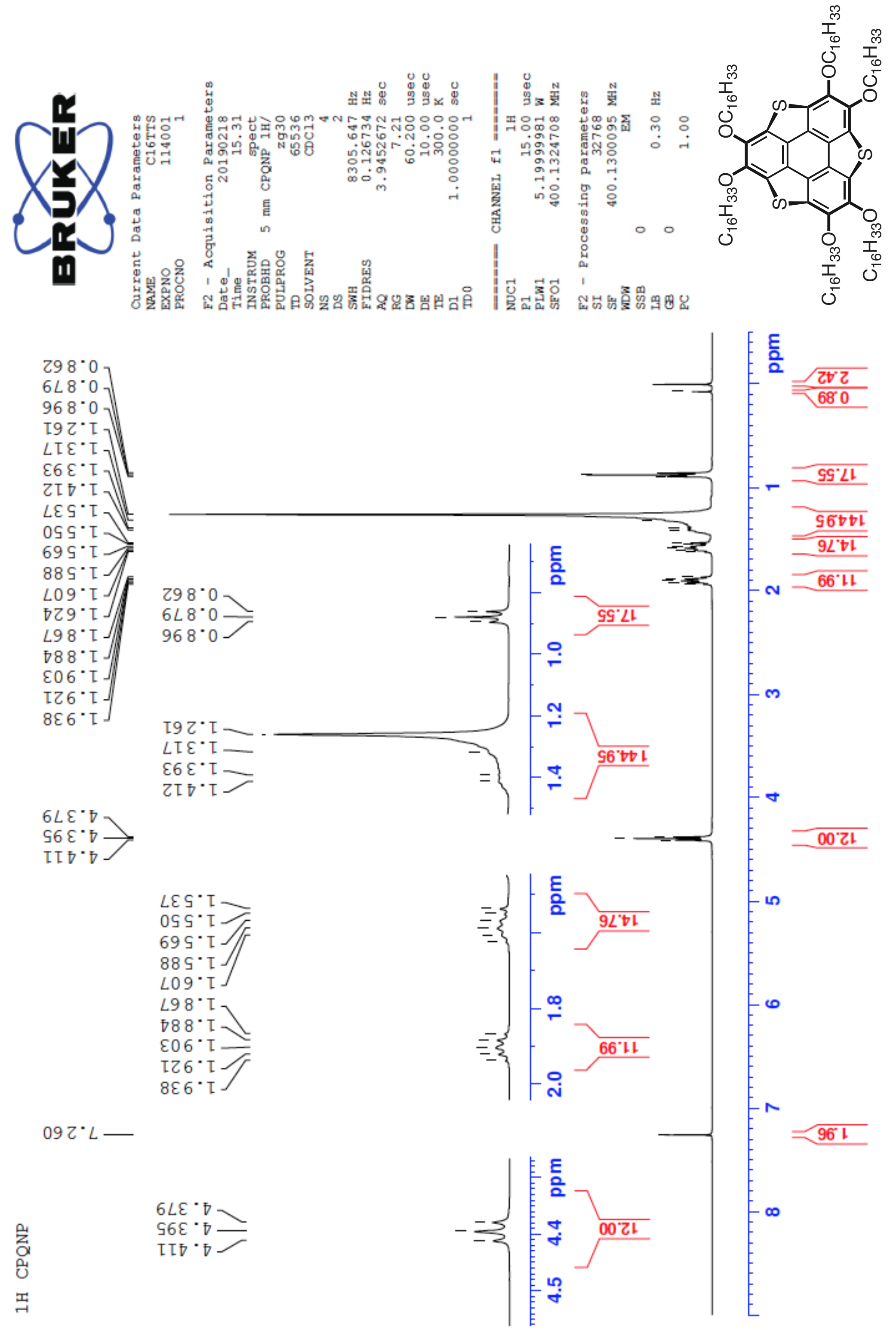

**$^{13}C$ NMR spectrum of C16SS in $CDCl_3$**

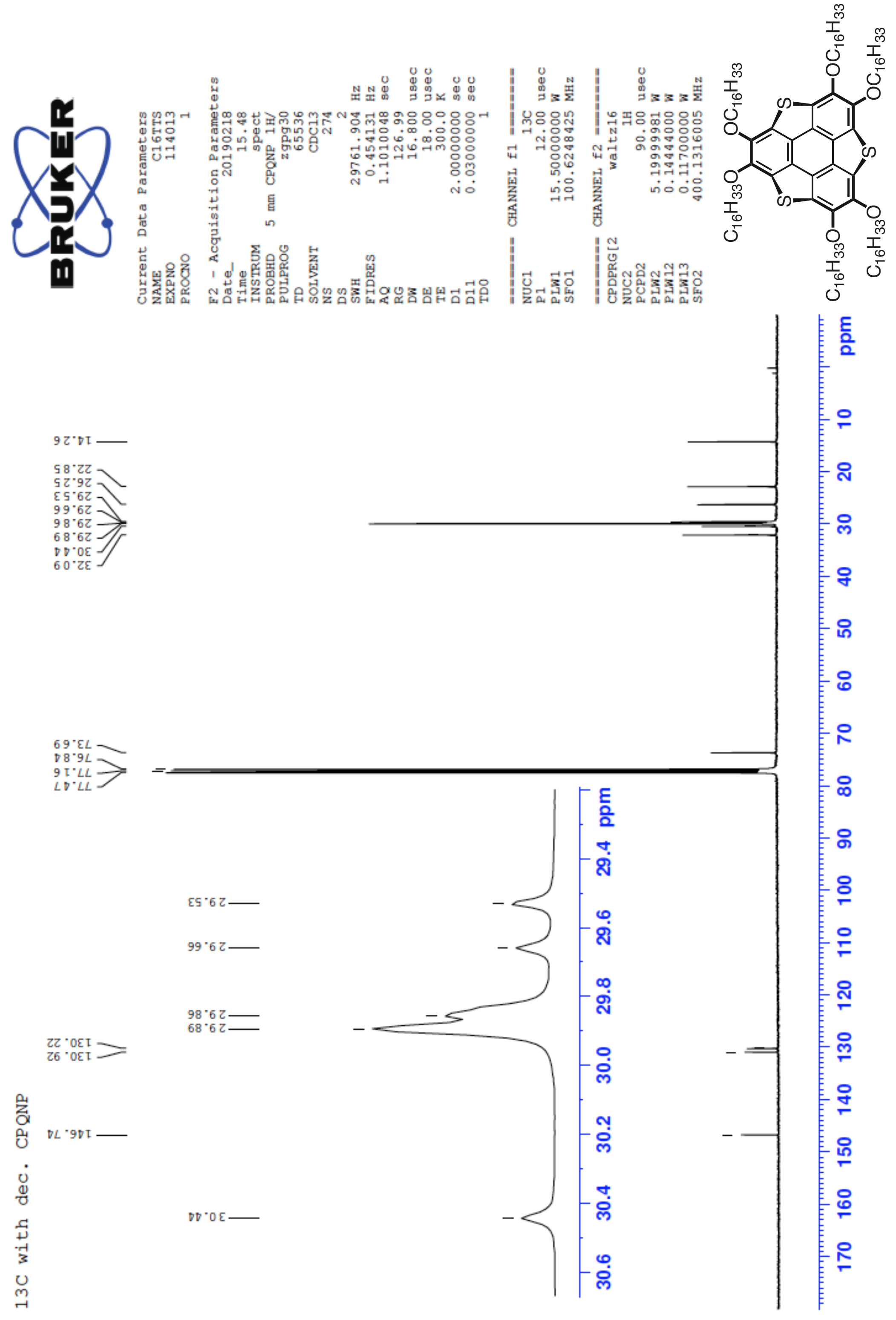


**DEPT-135 of C16SS in $CDCl_3$**

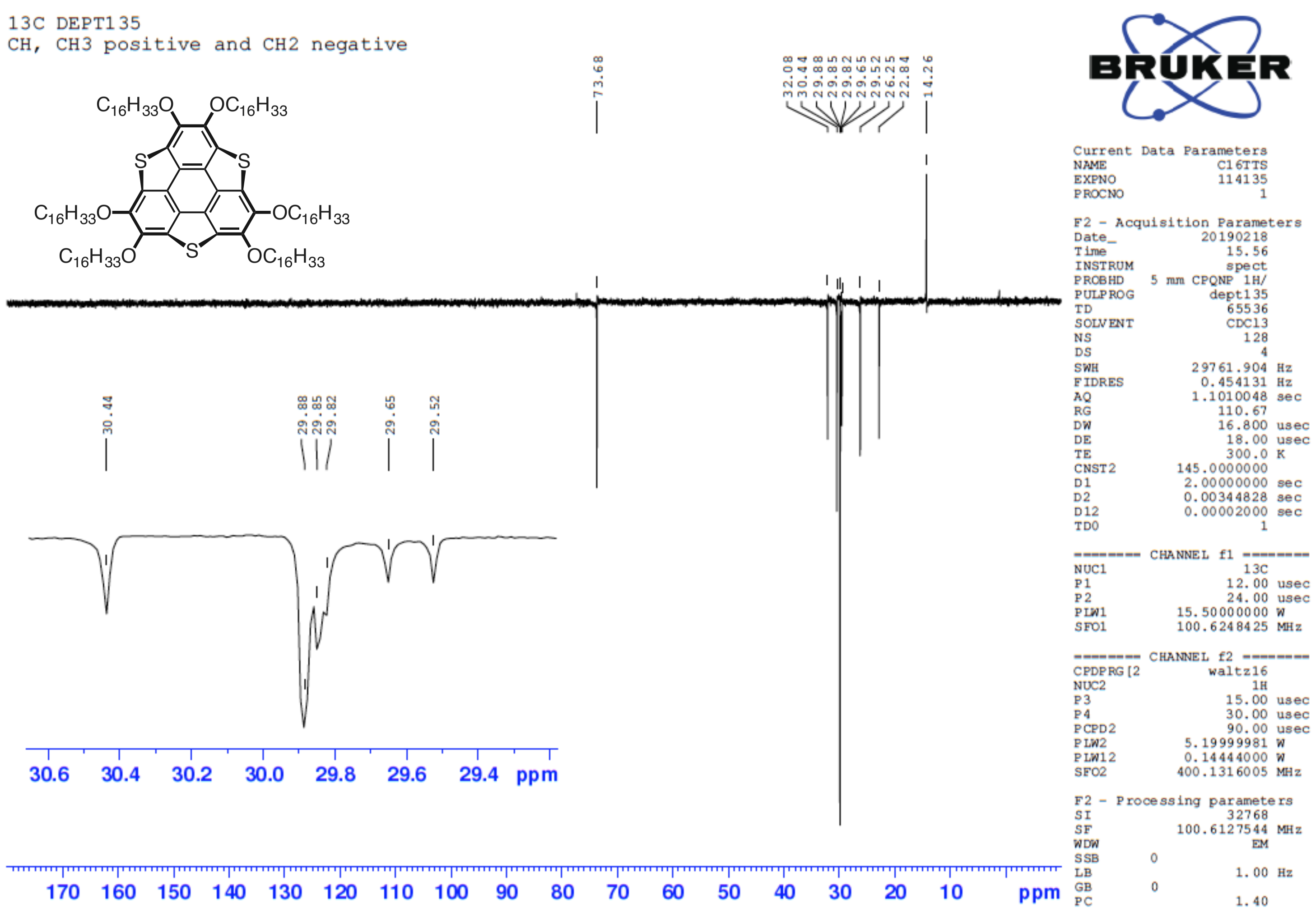
13C DEPT135
CH, CH3 positive and CH2 negative
$C_{16}H_{33}O$
$OC_{16}H_{33}$
S
73.68
32.08
30.44
29.88
29.85
29.82
29.65
29.52
26.25
22.84
14.26
30.6
30.4
30.2
30.0
29.8
29.6
29.4
ppm
170 160 150 140 130 120 110 100 90 80 70 60 50 40 30 20 10 ppm
BRUKER
Current Data Parameters
NAME C16TTS
EXPNO 114135
PROCNO 1
F2 - Acquisition Parameters
Date_ 20190218
Time 15.56
INSTRUM spect
PROBHD 5 mm CPQNP 1H/
PULPROG dept135
TD 65536
SOLVENT CDCl3
NS 128
DS 4
SWH 29761.904 Hz
FIDRES 0.454131 Hz
AQ 1.1010048 sec
RG 110.67
DW 16.800 usec
DE 18.00 usec
TE 300.0 K
CNST2 145.0000000
D1 2.00000000 sec
D2 0.00344828 sec
D12 0.00002000 sec
TD0 1
======== CHANNEL f1 ========
NUC1 13C
P1 12.00 usec
P2 24.00 usec
PLW1 15.50000000 W
SFO1 100.6248425 MHz
======== CHANNEL f2 ========
CPDPRG[2 waltz16
NUC2 1H
P3 15.00 usec
P4 30.00 usec
PCPD2 90.00 usec
PLW2 5.19999981 W
PLW12 0.14444000 W
SFO2 400.1316005 MHz
F2 - Processing parameters
SI 32768
SF 100.6127544 MHz
WDW EM
SSB 0
LB 1.00 Hz
GB 0
PC 1.40
